# Patient considerations in trauma-focused treatment decision-making: a qualitative study

**DOI:** 10.1186/s12888-026-08125-7

**Published:** 2026-05-02

**Authors:** Dionne M. Dopmeijer, Noortje I. van Vliet, Maarten K. van Dijk, Manna A. Alma

**Affiliations:** 1 Dimence Mental Health Group, Burgemeester Roelenweg 9, Zwolle, 8021 EV The Netherlands; 2 Dimence Mental Health Group, Pikeursbaan 3, Deventer, 7411 GT The Netherlands; 3 Department of Health Sciences, Applied Health Research, University Medical Center Groningen, University of Groningen, Hanzeplein 1, Groningen, 9700 RB The Netherlands

**Keywords:** PTSD, Qualitative study, Patient perspectives, Decision-making process, Psychotherapy, Shared decision-making.

## Abstract

**Background:**

Post-traumatic stress disorder (PTSD) is a common psychiatric condition, with a global prevalence of 3.9% and a lifetime prevalence of 7.4% in the Netherlands. Although numerous evidence-based treatments are available, their use varies considerably. Understanding patient perspectives and experiences in the decision-making process when choosing a specific treatment is crucial for improving the quality of care.

**Objectives:**

This study aimed to explore the decision-making process from the perspective of patients with PTSD, focusing on their considerations and experiences when choosing a specific psychotherapeutic treatment.

**Methods:**

In this qualitative study, twelve semi-structured interviews were conducted with patients with PTSD (aged 21–55 years) receiving care at a mental health organisation in the east of the Netherlands. Data were analysed using reflexive thematic analysis, informed by grounded theory–inspired analytic techniques. Analysis was iterative and inductive, focusing on the identification and interpretation of patterns of meaning across patients’ accounts.

**Results:**

Five themes were identified, connected through an overarching interpretive concept of patient-attunement: (1) the role of treatment characteristics (2), the role of therapeutic factors (3), the role of treatment duration and intensity (4), the role of significant others, and (5) the role of information about trauma-focused treatment. Patients emphasised the importance of personalised information and support from clinicians and significant others. Ongoing responsiveness within the therapeutic relationship, including trust and collaboration, was central to experiencing the decision-making process as supportive and meaningful.

**Conclusions:**

For patients with post-traumatic stress disorder, multiple factors play a role in choosing a psychotherapeutic trauma-focused treatment. This study shows that continued attunement to patients’ diverse needs is central to the decision-making process. Such attunement may take various forms, including attention to the therapeutic relationship, discussion of treatment characteristics and frequency, and responsiveness to patients’ preferences regarding information provision. Involving patients’ support systems may further support decision-making. Together, these findings suggest that clinicians should remain attentive to patients’ needs at multiple points during the pre-treatment phase. Approaches such as shared decision-making may help facilitate this attunement.

**Clinical trial number:**

Not applicable.

**Supplementary Information:**

The online version contains supplementary material available at 10.1186/s12888-026-08125-7.

## **Introduction**

Post-traumatic stress disorder (PTSD) is a psychiatric condition that can develop following exposure to a traumatic or highly stressful event. Globally, an estimated 3.9% of the population has experienced PTSD at some point in their lives [1]. In the Netherlands, approximately 80% of the general Dutch population reports having experienced one or more traumatic events involving actual or threatened death, serious injury, or sexual violence [2]. The lifetime prevalence of PTSD is estimated at 7.4%, making it one of the most common psychiatric disorders in the country [3].

PTSD is characterized by four symptom clusters: (a) repeated unwanted reliving of the traumatic event; (b) avoidance; (c) negative cognitions and negative affect; and (d) increased irritability [4]. The disorder is associated with significant health and societal burdens, including increased risks of suicide attempts and reduced quality of life [5]. Individuals with PTSD often experience comorbid physical health problems such as musculoskeletal pain, cardio-respiratory symptoms, and gastrointestinal issues [6]. Furthermore, PTSD is linked to education and employment difficulties, contributing to societal costs and profoundly impacting affected individuals [7].

There are several evidence-based treatments for PTSD including both psychotherapy and medication. Clinical practice guidelines for adults [8] and the Dutch guidelines for psychotrauma and stressor-related disorders [9] recommend psychotherapies such as Prolonged Exposure (PE), Cognitive Therapy (CT) and Cognitive Processing Therapy (CPT), Eye Movement Desensitization and Reprocessing Therapy (EMDR), and Trauma-Focused Cognitive Behavioural Therapy (TF-CBT). Other treatments, although less frequently studied, have also demonstrated effectiveness, including Brief Eclectic Psychotherapy for PTSD (BEPP), Narrative Exposure Therapy (NET), Writing Therapy, and Imagery Rescripting (ImRs) [8, 9].

Although several evidence-based psychotherapeutic treatments for PTSD are available, research indicates that some methods are used more frequently than others [10]. Van Minnen et al. [11] found that Imaginal Exposure (IE) is applied less often compared to EMDR. Their study suggests that limited training in IE may lead to concerns about symptom worsening, resulting in doubts about its effectiveness for multiple traumas. Hundt et al. [12] reported that provider characteristics, such as cognitive-behavioural orientation, younger age, fewer years of experience and more time spent treating patients with PTSD, were positively associated with the use of evidence-based psychotherapies, including cognitive processing therapy, prolonged exposure, and EMDR.

Regarding patients‘ choice of trauma treatment, the literature highlights diverse preferences and considerations. Several studies indicate that most patients prefer psychotherapy over pharmacotherapy [13–15]. Factors influencing these choices include the perceived rationale of the therapy, a strong need to talk about the trauma, beliefs about the treatment mechanism, and fear of side effects or symptoms worsening [13, 15, 16]. Patients’ assumptions also play a role, such as the belief that trauma should be addressed at its roots rather than symptomatically, the perception that professional help is necessary, and the expectation that psychotherapy offers better long-term outcomes than pharmacotherapy [17, 18]. Additional factors include prior successful treatment experiences that build confidence, familiarity with the clinician, and trust in the clinician’s competence and care [16]. Finally, encouragement from peers (e.g. veterans) and desperation for symptom relief have also been reported as reasons for choosing psychotherapy [16].

Research indicates that when patients are given a choice of treatment, their quality of life improves compared to situations where they receive treatment that is not their preferred option [19]. Furthermore, shared decision-making, defined as collaborative communication between patient and clinician on healthcare decisions, is associated with greater knowledge, higher treatment satisfaction, and symptom improvement [20]. In a review on shared decision-making for PTSD, Harik [21] emphasizes that preliminary evidence suggests this approach is promising, as involving patients in treatment decisions enhances engagement with evidence-based care, which in turn improves outcomes and cost-effectiveness. To enable such joint decision-making, it is essential to understand patients’ motivations and considerations, as these insights are critical for personalizing PTSD treatment.

Understanding patients’ considerations is essential for advancing patient-centred care and optimising clinical practice. Insight into these processes can inform psychoeducation, enhance shared decision-making, and support clinicians in tailoring treatment discussions. To address this gap, we conducted a qualitative study using reflexive thematic analysis to explore how patients with PTSD experience the decision-making process regarding psychotherapeutic treatment and to develop an interpretive understanding grounded in their reported experiences.

## Methods

### *Design*

We conducted a qualitative interview study using reflexive thematic analysis, as outlined by Braun and Clarke [22], to explore patients’ experiences with the decision-making process regarding a specific psychotherapeutic treatment. The analysis was informed by several grounded theory–inspired analytic techniques as described by Charmaz [23].

Semi-structured interviews were carried out with patients undergoing treatment for PTSD, or on a waiting list for PTSD treatment, across four different teams (two teams specialised in Anxiety & Mood Disorders and two in Personality Disorders) of the Dimence Mental Health Organisation, located in the east of the Netherlands. The consolidated criteria for reporting qualitative health research (COREQ) were applied [24].

### *Ethical considerations*

The Medical Ethical Review Board of the University Medical Center Groningen determined that the Dutch Medical Research with Human Subjects Law did not apply to this study (reference number METc 2022/054). Written informed consent was obtained prior to the interview. Patients agreed to audio recording of the interviews and their use for scientific research after pseudonymisation. Confidentiality was emphasized, and patients were informed of their right to withdraw at any time.

### *Participants and sampling*

Patients diagnosed with PTSD were eligible if they met the following criteria: (a) age 18–65 years, (b) PTSD diagnosis according to DSM-5, (c) currently receiving PTSD treatment or on a waiting list, and (d) comorbidity allowed if PTSD was the primary focus of treatment. Convenience sampling was used to select a wide variety of patients through four teams of the Dimence Mental Health Organization. Clinicians identified eligible patients and obtained permission to share their contact details with the research team. Patients received an information letter and were contacted by the lead researcher (DD) for eligibility screening and further explanation of the study. Of 15 contacted patients, 12 agreed to participate; 3 declined due to lack of interest or personal circumstances.

A total of 12 patients was included in the study. In line with a reflexive thematic analytic approach, sample size was considered sufficient on the basis of the information richness of the data, the depth and diversity of patients’ accounts, and their relevance to the research question. Data collection and analysis proceeded iteratively, allowing for ongoing assessment of whether the dataset offered sufficient depth and nuance to support a meaningful interpretive analysis. After 12 interviews, the data were judged to provide a coherent and conceptually rich basis for identifying and interpreting patterned meanings across participants’ accounts. The sample size was therefore considered appropriate for the analytic aims of the study.

### *Data collection*

Semi-structured interviews were conducted using an interview guide. The interview guide was primarily informed by the clinical experience of the research team with patients with PTSD. These experiences shaped the selection of broad topic areas, while questions were used flexibly and did not determine the analytic outcomes. Topics included experiences of receiving a PTSD diagnosis, considerations during treatment decision-making, sources of information about treatment forms, the role of clinicians and relatives, and recommendations for improving the decision-making process. Interviews were conducted between November 2022 and August 2023, mostly face-to-face at Dimence offices (*n* = 8) or patients’ homes (*n* = 3), and one online via MS Teams. Interviews lasted 30–45 min and were audio recorded. Patients completed a short demographic questionnaire (gender, age, marital status and educational level), race or ethnicity was not systematically assessed.

Interviews were conducted by DD (female, MSc, licensed healthcare psychologist in training to become a clinical psychologist), who had no prior relationship with the patients. Field notes were taken after each interview. One interview was conducted with the assistance of a professional interpreter. While this facilitated participation, the use of an interpreter may have influenced nuances in expression and interpretation and was therefore considered during analysis.

### *Analysis*

Data were analysed using reflexive thematic analysis, as outlined by Braun and Clarke [22], informed by several grounded theory–inspired analytic techniques by Charmaz [23]. The analysis followed a cyclical and iterative process, with ongoing movement between data familiarisation, coding, and theme development. Interviews were transcribed verbatim and analysed using Atlas.ti (version 23.4.0.29360).

Initial inductive coding generated 47 preliminary codes, which were iteratively refined through close engagement with the data. Constant comparison was used throughout the analysis to explore similarities and differences across patients’ accounts and to support analytic sensitivity. Analytic memo writing was employed to document interpretive insights and reflexive observations throughout the process.

Through iterative analysis and reflexive discussion within the research team, five themes were developed as patterns of shared meaning organised around a central idea, consistent with reflexive thematic analysis. In the later stages of analysis, attention focused on how the themes related to one another at a more abstract level, allowing for the development of an overarching interpretive concept that articulated their conceptual coherence.

To support analytic quality, the first three interviews were independently coded by two researchers (DD and MA; female, PhD, social scientist), followed by collaborative reflexive discussion to compare interpretations and reflect on analytic decisions. These discussions aimed to deepen reflexivity rather than establish coding consensus or reliability. Interpretive decisions and developing themes were further discussed in regular research team meetings (DD, MA, MD (male, PhD, licensed clinical psychologist), and NV (female, PhD, licensed healthcare psychologist in training to become a clinical psychologist), providing opportunities for critical dialogue and reflexive engagement with the data.

Illustrative quotes were translated from Dutch into English and reviewed by the research team to ensure conceptual and contextual accuracy.

### *Researcher reflexivity and positionality*

The lead researcher (DD), a licensed healthcare psychologist in training to become a clinical psychologist, conducted all interviews. Her professional background and experience in delivering trauma-focused therapy may have influenced data collection and interpretation. Reflexivity was maintained through analytic memos and regular team discussions to address potential biases. The research team comprised an interprofessional group with diverse disciplinary backgrounds, including a healthcare psychologist (in training to become a clinical psychologist) and researcher (NvV), a clinical psychologist and researcher (MvD), and a researcher with expertise in qualitative methods and applied health sciences (MA). Several team members were practicing psychologists with prior experience providing trauma-focused treatment, while others contributed methodological and theoretical expertise. This diversity informed the study’s design, interpretation, and analysis and was continuously reflected upon throughout the research process.

### *Analytic quality*

Analytic quality was supported through reflexive and transparent practices consistent with reflexive thematic analysis. Throughout the analytic process, memos were used to document interpretive decisions and reflexive considerations during coding and theme development. Regular collaborative analytic discussions within the research team provided opportunities to critically examine emerging interpretations, challenge assumptions, and refine analytic insights. Multiple researchers engaged with the data from different disciplinary perspectives to enrich interpretation and support reflexive sense-making. Member checking was not conducted, given the sensitive nature of the topic and concerns about patients’ burden. Instead, analytic rigour was supported by careful attention to the accuracy and contextual integrity of illustrative quotes, which were collaboratively reviewed by the research team following translation from Dutch into English.

## Results

Twelve patients participated in this study, including one male. The mean age was 32 years. Most patients were of Dutch origin (*n* = 10), and two were of non-Dutch origin (*n* = 2); all were permanent residents of the Netherlands. Table 1 gives an overview of the patients’ characteristics.

**Table 1:** Characteristics of interviewed patients (*N* = 12)

Characteristic	*N*
***Age***	
*Mean*	*31.9 (SD = 9.57)*
* Range*	*21–55*
***Gender***	
* Male*	*1*
* Female*	*11*
* Nationality*	
* Dutch*	*10*
* Non-Dutch*	*2*
***Highest attained education level***	
* High school*	*1*
* Vocational education*	*6*
* Bachelor’s degree*	*3*
* Master’s degree*	*2*
***Household status***	
* Single*	*2*
* Cohabiting*	*3*
* Married*	*5*
* Divorced*	*1*
* Widow*	*1*

Information on nationality was collected; however, race or ethnicity was not systematically recorded

**Fig. 1:**
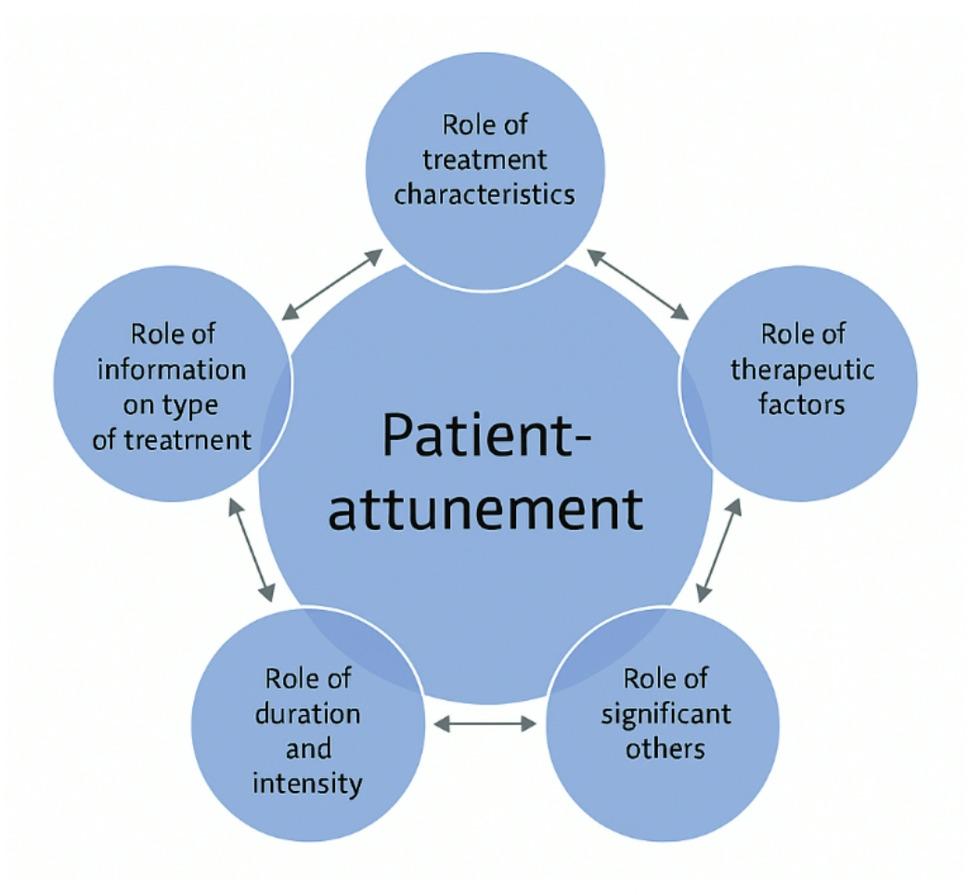
Interpretive overview of themes connected through patient-attunement

Analysis of the interviews resulted in an iteratively developed set of inductive codes, refined through close engagement with the data and reflexive analytic discussion. Through this process, five themes were identified that captured patterned meanings across patients’ accounts (Fig. 1). Across these themes, a shared pattern of meaning emerged, which we conceptualised as an overarching interpretive concept: patient-attunement. In this study, patient-attunement refers to clinicians’ ongoing responsiveness to patients’ individual needs, preferences, and circumstances during treatment decision-making. It encompasses relational attunement within the therapeutic relationship, sensitivity to patients’ informational needs, and adaptation of treatment discussions to patients’ practical and social contexts.

The five related themes are: (a) the role of treatment characteristics, (b) the role of therapeutic factors, (c) the role of significant others, (d) the role of treatment duration and intensity, and (e) the role of information about type of treatment for PTSD. During the interviews, patients also offered reflections and suggestions regarding how clinicians might better support treatment decision-making. The recommendations for clinicians to improve the decision-making process are discussed within the paragraphs that describe these themes.

### Theme 1: The role of treatment characteristics

Treatment characteristics, often described as an explanation of the principles of a specific psychotherapy, played an important role in selecting a specific treatment. Several patients expressed the need they felt to talk about their issues and selected a type of treatment that addresses these issues.

*‘I have no problem seeing images in front of me*,* I see them very often. I just try to place it all and understand it. I want to have more of a dialogue about it and how can that be*,* you know? So then I thought*,* I’ll have a lot more use for it if I just talk to someone about it. And then also… it’s not one image*,* but my whole life*,* so it has to be placed as well. So that’s why I thought this [specific treatment form] was better’. (Female*,* 30s)*

Other patients mentioned that they are ‘visual thinkers’ and preferred a type of treatment that connects to this. In addition, the amount of homework and time (outside the sessions) was a reason for choosing (or not choosing) a type of treatment. Another factor considered was the extent to which talking about fearful or anxious memories was necessary and if there was a high probability of avoidance (during the session).

*‘Because we kept going back to the memory a lot*,* that was much better for me. And*,* uhm yes*,* we repeated this memory maybe six or seven times. and that*,* I can say*,* uhm*,* and then I was completely done with the memory. So that’s why*,* yes … I had a slightly better feeling with EMDR’. (Female*,* 30s)*

Some patients had already tried different forms of treatment in the past which were insufficient and now wanted to try other forms of treatment with different characteristics. One patient mentioned the importance of the treatment being evidence-based. Also resumption of treatment which was successful in the past was a reason to choose this form of treatment, as the following quote displays:

*‘It was purely for me because I already know it [specific treatment]*,* and I knew what was expected of me’. (Female*,* 20s)*

### Theme 2: The role of therapeutic factors

Important aspects of the therapeutic relationship (between the clinician and patient) were proper communication, honesty, empathy, motivating others, cooperation (alliance) and trust. Trusting the clinician and the entire treatment team and following their advice played an important role in the decision-making process. Patients described trust as a process of feeling understood, being taken seriously and being seen and heard by the clinician.

*‘And that they really listened to me*,* and I truly had the feeling*,* the idea*,* that finally*,* there was someone who was going to help me. Someone with whom I could be vulnerable*,* knowing that it’s okay and that I don’t have to do it alone.’ (Female*,* 30s)*.

*‘I can say*,* yes*,* that the support of the clinician is very important for the patient’. (Female*,* 30s)*

Furthermore, patients indicated the importance of keeping all treatment options open for the patient to choose; they recommended not to steer in one direction, to show courage and give patients time to think about their options in between sessions. Patients emphasized to stay open to and consider the patient’s individual circumstances.

*‘Uhm*,* don’t let someone make a decision on the same day*,* just give it 48 hours. Let them talk about it at home*,* think it over*,* and let it sink in. Provide multiple options*,* offer leaflets*,* uhm and then schedule a new appointment to discuss it again and let them make their own choice.’ (Female*,* 20s)*.

*‘Uhm*,* well*,* they always left the choice up to me*,* which I found the most pleasant. Of course*,* they laid out all the options and gave their honest opinion and advice*,* but it was always up to me what I would do. Yes*,* actually*,* it has always been my own decision. Of course*,* I relied on the advice they gave me*,* but it’s not like they ever forced a choice on me or anything. I always knew the ball was in my court*,* and they also said*,* “We can help you*,* but you have to do it yourself.’ (Female*,* 20s)*.

Moreover, patients emphasized the importance of going through the process together and offering support, hope and perspective on the future.

*‘I think a lot of people with eh trauma -including myself- feel like they have to carry it alone. But if you’re lucky enough to have a good psychologist or clinician*,* I think you’re fortunate to be able to go through a little part of it together*,* alongside someone else.’ (Female*,* 50s)*.

### Theme 3: The role of significant others

This theme consists of two subthemes: the role of family and friends and the role of clinicians, which are both described below.

### Role of family and friends

All patients informed someone from their own network that they were starting the treatment. Patients said that consulting family members could either lead to deciding based on positive experiences with a treatment or deter them from selecting a treatment based on negative experiences. Some patients included the opinion of their family and friends in the decision-making process, whereas others mainly informed their network.

*‘My sister had it too*,* so*,* I just heard really good experiences.’ (Female*,* 40s)*.

### Role of clinicians (healthcare professionals)

Several patients mentioned that the ideas of their clinician played a role in their choice of a specific treatment. The clinician’s experience and expertise in PTSD treatment was leading in this. Some also involved their outpatient counsellor, and found this to be helpful in the decision-making process. One patient said that the opinion and clinical experience of the General Practitioner (GP) also weighed in making a final choice and selecting a certain form of treatment.

*‘Initially*,* I would have chosen something else*,* but that was the easy way out. Uhm*,* and this is actually the most difficult for me*,* but it’s probably going to have the most effect in the long term. Uhm*,* so I followed the advice of my clinician in that’. (Female*,* 20s)*

### Theme 4: The role of duration and intensity of the treatment

All patients stated that the duration of treatment (from the beginning to the end of the treatment in months) was influential but was not the decisive reason for selecting a specific treatment. Most of the patients felt that the total duration (in months) was secondary to other considerations. Some mentioned that it was helpful to have an estimate of the duration of the treatment, while others mentioned that this could also cause a lot of pressure during the treatment.

*‘… How long it takes doesn’t matter much to me*,* as long as it brings peace of mind for myself’. (Female*,* 20s)*

Regarding the intensity of the treatment (frequency of sessions per week) a majority of the patients preferred one session a week. The main reason was that the sessions could be intensive and time was needed between sessions to recover from this, according to patients.

*‘Yes*,* I always thought once a week would be enough for me. I thought*,* because I can handle it… and I do… but*,* you know.*,* physically*,* it takes a lot.’ (Female*,* 30s)*.

Some patients either preferred two sessions per week, or they did not have a preference. One patient specified the need for more sessions (preferably a session per day), while another patient mentioned to prefer a session once every two weeks or once a month. A few patients also mentioned that waiting lists played a role in choosing a specific treatment.

*‘I had signed up for both*,* and whichever came first*,* I was just going to do that.’ (Female*,* 20s)*.

Some patients recommended the opportunity to have additional contact with their clinician in between sessions. The importance of exploring the options, and/or frequency, of contact with the clinician was emphasized, especially when a patient is on a waiting list, awaiting to start treatment.

*‘Maybe it’s an idea - I have no idea if that’s possible - but to have a phone contact in between*,* I don’t know*,* eh*,* to talk to someone.’ (Female*,* 30s)*.

Also more practical reasons were cited for choosing treatment, such as short travel time to the location, being able to keep working and financial circumstances.

*‘I did think about it*,* uhm*,* because I actually assumed it was once a week*,* and that’s fine. But if it were more often*,* I’d have to see if that was possible with work’. (Female*,* 20s)*

*‘Uhm*,* well*,* I have to be very considerate of others*,* and when I’m in [place of residence]*,* I have to consider the psychologist’s schedule*,* my mother’s schedule who brings me*,* and my friend’s schedule who then picks me up again… so that’s not really doable at any given time.’ (Female*,* 20s)*.

### Theme 5: The role of information about the type of treatment

Most patients reported that they felt it was (somewhat) important to be well informed of evidence-based types of treatment.

*‘Um*,* still pretty important*,* I think. Um*,* but that’s just because I want to have some idea of what I’m getting into*,* what to expect. So that I’m not constantly in the process of ‘yes*,* okay*,* but what happens now*,* and what comes next?’ That I already have some idea.’ (Female*,* 20s)*.

One patient indicated limited need for information and emphasized the desire to be cured of symptoms regardless of the treatment method.

*‘Information or no information*,* that doesn’t matter much to me … the important thing is that I recover.’ (Male*,* 20s)*.

Information on the type of treatment was provided in various ways, through leaflets, brochures, websites and online modules. Several patients expressed that they looked up information themselves after the advisory sessions with the clinician. Some patients mentioned that the information provided in the session with the clinician was sufficient, and they did not feel the need for an additional leaflet or brochure. The importance of accessible language in the leaflets was emphasized, so that everyone can understand it.

*‘I really liked the fact that it was dealt with when I was there*,* just briefly*,* that the information I needed was explained*,* uhm*,* by the psychologist. Everything I read in that brochure*,* I’ve actually forgotten; it was so difficult to read*,* and you still didn’t really have an idea of what it was like. But the way they talked about it when I was there or on the phone… I really liked that’. (Female*,* 20s)*

Being able to ask the clinician additional questions was felt to be helpful. The timing (in the decision-making process) of providing information was seen as best during the intake procedure (first sessions) or at the start of the actual treatment by the clinician. Also mentioned was the option of self-indicating the need for (additional) information during the selection process. Various patients suggested that it could add value to the process of providing information on treatment forms, to display a video or short story by an expert by experience (peer coach). Also, an instruction video of the specific treatment could be helpful to gain more insight in how the treatment is conducted.

*‘That you can work with certain people who have already completed the treatment*,* that they make a recording with their own story*,* tell their own experience with the treatment*,* and that*,* if you give permission for that*,* of course*,* it can be used for new patients who are going to undergo the treatment.’ (Female*,* 20s)*.

### Overarching interpretive concept

What emerged from the data is that patients expressed highly diverse needs and preferences regarding the decision‑making process, a pattern that recurred across all identified themes. The themes are connected through patients’ personal needs and wishes, which differed substantially between individuals. We conceptualised this shared pattern of meaning as patient‑attunement, referring to clinicians’ ongoing responsiveness to patients’ varying needs and wishes. According to patients, such responsiveness was central to experiencing the decision‑making process as supportive and meaningful.

## Discussion

In this qualitative study, the perspectives and experiences of patients with PTSD regarding the decision-making process for a type of trauma treatment were explored. Five themes emerged from the data analysis: the role of treatment characteristics, the role of therapeutic factors, the role of significant others, the role of duration and frequency and the role of information on a type of treatment. All themes are connected through the overarching interpretive concept of patient-attunement.

Patients mentioned that treatment characteristics played a role in their decision-making process, which is in line with previous studies that also found that characteristics of treatment and a strong need to talk about the trauma are important for patients when choosing a type of treatment [13, 15, 17, 18]. Previous treatment experiences also played a role, as it could increase patients’ confidence in their ability to handle psychotherapy [16]. Finsrud et al. [25] in their study on therapeutic relationship factors, describe the importance of trust in treatment, referring to patients’ experience that treatment is a meaningful solution to their problems and provides positive expectations for improvement. This is consistent with our study, in which patients described that characteristics of treatment play an important role, for example, the need to talk about their problems and choose a treatment approach that addresses these concerns. Contrary to the findings of Zoellner et al. [15], our study did not identify fear of side effects as an important factor in treatment choice. This may be because our study focused exclusively on psychotherapy interventions rather than pharmacological treatments.

Our study found that therapeutic factors such as communication, honesty, empathy, and trust were considered highly important aligning with findings of Hundt et al. [16]. A study by Nissen Lie et al. [26] on therapist predictors of early work alliance, found that, among other things, professional self-doubt - otherwise described as an attitude of therapist humbleness and sensitivity- seems to facilitate alliance development. In our study, patients described similar key therapist characteristics, such as honesty, empathy, and trust, as important factors. Similarly, Finsrud et al. [25] identified key therapeutic relationship factors as confidence in the therapist’s qualities, including the ability to understand, help, and collaborate with the patient. This corresponds with our findings, where patients emphasized the importance of going through the process together, receiving support, and gaining hope and perspective for the future.

Several patients mentioned that the ideas of their clinician also played a role in their choice for a specific treatment. The clinician’s experience and expertise in PTSD treatment was leading in this. These findings are consistent with findings in the study of Hundt et al. [16] that prior knowledge about and familiarity with the clinician and knowing that the clinician was a competent caring individual played an important role for patients. A study by Schladitz et al. [27] on the experiences of shared decision-making among people with mental illness and their family members found that both groups have a strong desire to be involved in treatment decisions and to participate in the diagnostic process. The authors also emphasized that the shared decision-making process should begin at the diagnostic phase. These findings align with the results of the present study, in which patients reported that consulting family members influenced the decision-making process regarding PTSD treatment. Some patients included the opinion of their family and friends in the decision-making process, whereas others mainly informed their network without actively involving them.

Regarding duration, frequency and intensity, we found that there were considerable differences in intensity preferences based on individual needs. We also found that treatment duration was influential but not the decisive factor in selecting a treatment. Most patients in this study preferred one session per week. These findings are notable given the increasing availability of inpatient treatments, which offer the possibility of multiple sessions per week. This preference may be explained by the specific group of patients we interviewed within one context.

Finsrud et al. [25] also highlighted the significance of therapeutic skills such as providing psychoeducation, explaining the therapeutic model, and connecting the model to the patient’s specific problems while providing hope and empowerment. These findings align with our findings that continuous personalization and adapting of information and communication skills are essential in meeting patients’ specific needs on how to receive information.

Our main finding is that continuous attunement is needed in the patient-clinician relationship. This means that throughout the decision-making process, clinicians should continuously discuss treatment preferences, including duration and frequency, and that they should adapt the way of providing information to match patient’s needs. Furthermore, it is important to involve the patient’s support system where possible and pay attention to the therapeutic relationship. These findings align with existing research on the importance of therapeutic relationship in treatment. While different psychotherapeutic frameworks may define the therapeutic relationship in varying ways, its significance is widely recognised [25, 26, 28]. More attention could be given to the therapeutic relationship during the pre-treatment phase. One way to implement this is through shared decision-making.

In the mental health sector, much attention has been paid to the importance of shared decision-making. Research has shown that shared decision-making positively influences patient satisfaction and health outcomes [29]. When properly implemented, it reduces decision ambivalence and improves treatment outcomes [30]. A widely used model for implementing shared decision-making in clinical practice is described by Elwyn et al. [31]. This model consists of three key steps. The first is choice talk, ensuring that patients are aware of the available treatment options. The second step is option talk, providing detailed information about each option. And the third step is decision talk, considering patient preferences and making a shared treatment decision [31]. For patients with PTSD, this model can be applied by first informing them that a decision needs to be made and ensuring they are involved to the extent they are comfortable (choice talk). Option talk involves sharing clear and comprehensive information about treatment options, using tools such as brochures, videos, or online resources. Finally, decision talk entails exploring the patient’s preferences and determining what is most important to them [21].

Patients in our study found it helpful to follow their clinician’s advice when it was properly discussed, aligning with the principles of shared decision-making and patient attunement. Shared decision-making is described as existing on a continuum [32]. Within this continuum we can allow different patients and variable situations, where sharing can at some point encompass most or some decisions.

### Strengths and limitations

This study provided patients with PTSD with the opportunity to share their experiences and express what they find most important in the decision-making process for treatment. These insights are valuable for healthcare professionals in tailoring care to patients’ needs and preferences. The qualitative data collected allowed for in-depth exploration of diverse patient perspectives. However, certain limitations must be acknowledged. First, the sample size was relatively small, which is inherent to qualitative research but may influence the transferability of the findings. In addition, the patients interviewed were predominantly women, resulting in limited representation of men. Additionally, the number of migrant patients was limited. Race or ethnicity was not systematically assessed, which limits insight into the cultural and racial diversity of the sample. Cultural differences may influence treatment decision-making, and this lack of diversity may restrict the transferability of the findings to more culturally diverse populations. An additional limitation is that detailed information about the characteristics of patients’ traumatic experiences was not known. While the content of the trauma was not systematically assessed, other relevant factors were also unknown, including the duration of the trauma (e.g., single-incident versus prolonged or repeated trauma) and the extent to which trauma-related symptoms interfered with daily functioning. These factors are known to influence symptom presentation, treatment needs, and treatment preferences, and may therefore have played an important role in patients’ decision-making processes. The absence of this information limits the ability to fully contextualise patients’ treatment choices and may affect the interpretation and transferability of the findings. Given that data were collected within one specific context, the transferability of findings is limited. Future research should explore diverse groups and settings to gain a broader understanding of considerations in the decision-making process.

### Implications

Our study findings have important implications for clinical practice. Clinicians are encouraged to adopt a flexible approach, recognising that patients’ preferences vary widely. Continued patient-attunement appears central to supporting informed decision-making, alongside a diverse range of resources to support informed decision-making. Clinicians should be mindful of the importance of the therapeutic relationship, as well in the lead-up to treatment. Clinicians can tailor their approach to meet patients’ needs by discussing the preferred methods, forms, and timing for providing information. To achieve this, it is essential for clinicians to have access to various psychoeducational resources. Additionally, clinicians must possess a thorough understanding of the rationale behind various treatment forms and be able to effectively communicate this to patients. It is also important to involve significant others in the process, inviting them to appointments when appropriate and desired by the patient. Throughout this process, clinicians should discuss preferences regarding the therapeutic relationship with their patients, such as offering expertise and advice, a sense of equality, and feelings of support and hope. Approaches such as shared decision-making may help clinicians translate patient-attunement into everyday practice, as these approaches focus on dialogue and exchange between patient and clinician. Given the study’s limitations, future research should explore decision-making preferences among diverse patient groups in different clinical contexts. The main implications are summarised in Table 2 to support their application in clinical practice.

**Table 2:** Clinical implications of patient-attunement in PTSD treatment decision-making

Theme	Clinical implications
Treatment characteristics	Explore how treatment principles, demands, and prior experiences align with individual patient preferences and coping capacity.
Therapeutic factors	Invest in the therapeutic relationship during decision-making by listening, offering support, and allowing time to consider options.
Significant others	When appropriate and desired, acknowledge and involve significant others to understand patients’ decision-making context.
Duration and intensity	Discuss treatment duration, intensity, and practical demands in relation to patients’ daily lives and resources.
Information provision	Tailor the timing, format, and depth of information to patients’ preferences using clear, accessible communication.
Overarching concept: patient-attunement	Continuously attune to patients’ needs and preferences; shared decision-making can support this in clinical practice.

## Conclusion

This study highlights the diverse perspectives of patients in the decision-making process for trauma-focused treatment. Healthcare professionals should focus on patient-attunement during this process, recognising that individual needs and preferences vary. Attention to the therapeutic relationship, discussing of treatment characteristics and frequency, and tailoring of information provision are all key elements. Involving patients’ support systems and applying shared decision-making may further enhance this process. Together, these practises can foster a sense of joint responsibility, strengthen trust, instil hope, and reinforce the experience of going through the process together.

## Supplementary Information

Below is the link to the electronic supplementary material.

Supplementary Material 1

## Data Availability

The data that support the findings of this study are available from the corresponding author (DD) upon reasonable request. The data are not publicly available because they contain information that could compromise the privacy of the research participants.
