# Supplementary material for: Patient considerations in trauma-focused treatment decision-making: a qualitative study
Source: BMC Psychiatry. 2026 May 2;26:490. doi: 10.1186/s12888-026-08125-7 (PMC13312743; doi:10.1186/s12888-026-08125-7)
Supplement: Supplementary file 1 — Supplementary Material 1 [file 12888_2026_8125_MOESM1_ESM.docx]

# **Appendix I: Interview Topic List**

*Preparations*

- Answering questions
- Signing Informed Consent
- Setting up equipment
- Testing equipment

*Introduction*

- Introduction to interview and interview topics
- Information will be kept confidentially
- Information is not traceable to individuals

*Patients’ experiences*

- Could you tell me the story from the first moment you received the diagnosis PTSD, how did this process go?
- Had you previously received this diagnosis in treatment, or was this the first time?
- What was it like hearing that you have PTSD? How do you reflect on this conversation?
- How do you look back on this conversation?
- Are there things you missed in this conversation?
- Are there things you would have liked more attention paid to?

*Decision-making process*

- Could you tell me what kind of treatment you are receiving or will be receiving?
- Could you explain how you made the decision for a specific treatment?

*Considerations in the decision-making process*

- Why did you choose this specific treatment?
- Which form of treatment appealed to you the most?
  - Why?
  - What did you focus on?
- Which form of treatment appealed to you less or not at all?
  - Why not?
  - Did you consider other forms of treatment? Which ones? Why?
  - What was the decisive reason for choosing the treatment?
- What role does the duration and frequency of treatments play in your considerations?
  - What would be an ideal frequency?
  - What would be an ideal duration?
- Did you also look for treatment options outside the Dimence Mental Health Organization?

*Information*

- Could you tell me what kind of information you received about PTSD treatments after the diagnosis?
- How important is it for you to be well informed about evidence-based treatments for PTSD?
- From whom did you receive this information?
- Role of the clinician?
- How did you experience this process of receiving information?
  - What went well?
  - What could be improved?
  - Did you feel the need for additional information after this conversation? For example, a brochure, website, or an overview?
- When would you have liked to receive information about treatments?
  - During the advisory sessions / after intake procedure?
- What information did you miss?
- Would you have liked to receive different information?

*Role of the clinicians and others*

- Who did you talk to about your choice?
  - Therapist
  - Family/friends
- What role did the therapist play in the decision-making process?
  - How did this influence your decision-making?
  - How did you experience this collaboration (motivation/trust)?

*Reflection*

- Looking back at the decision-making process:
  - What went well?
  - What could be improved?
  - Did you feel the need for additional conversations in this decision-making process?
- What tips do you have for professionals when it comes to the decision-making process?
- What specifically would you like to see different or add to this process?

*Conclusion*

- Do you have any questions or comments regarding the topics we discussed?
- Explanation of the continuation of the study: time available for any questions on the study.
- Thank you very much for participating in the survey.
